# A Cure for Car Sickness

**Published:** 1881-06

**Authors:** 


### A CURE FOR CAR SICKNESS.

Many persons, especially ladies, are great sufferers from that form of nausea and headache known as "car sickness." A journey by rail has for them all the discomfort and suffering that an ocean voyage has to the majority of travelers. A lady who had occasion to take a short trip on the Lowell road—and she never travels by rail for pleasure—was, as is usual with her, as thorougly sick as ever a landsman is on the "heaving deep," by the time she had ridden a dozen miles. The conductor of the palace car, who was apparently very familiar with such cases, told the sufferer's companion that a sheet of writing paper, worn next to the person, directly over the chest, was a sure preventive of the trouble in nine cases out of ten. He had recommended it to hundreds of travelers, and rarely knew it to fail. The prescription seemed very like a "charm"—a horsechestnut carried in the pocket to ward off rheumatism, or a red string around the neck to prevent bleeding at the nose. But it was simple, and could at least do no harm. For the return trip, a sheet of common writing note-paper was fastened inside the clothing as directed. Result—a perfectly comfortable journey, without a hint of the old sickness that had for years made travel by rail a horror.—*Boston Herald.*

### SANITARY DEPARTMENT OF "HALL'S JOURNAL OF HEALTH."

We are daily receiving requests from our readers to purchase for them "HEALTH FOODS," "FOOD MEDICINES," PURE BAKING POWDERS, and various MEDICINES that our experience has taught us the value of; also standard books; and last but not least, many families rely on us to procure for them PURE BOVINE VACCINE MATTER, which is the only vaccine matter that is always safe. These commissions already keep one person employed much of the time. We have therefore determined to make this a branch of our regular business, and announce ourselves ready to fill orders for GLUTEN of WHEAT, GLUTEN COFFEE, COOKED FOODS, as CRUSHED WHEAT, BARLEY, OATS, CORN and RYE, also the various preparations of MALT, and for books, medicines, vaccine matter and any other like articles that can be found in New York market.

E. H. GIBBS & CO.

### THEN AND NOW.

The progress made in the manner of conveying news during the last 20 or 30 years is something remarkable. Intelligence in the old days traveled by a process so slow that it amuses us now to hear of it. The historian relates that when the battle of Waterloo was fought and the dispatches three days afterward reached London, they were printed in newspapers and the newspapers loaded into mail coaches. By day and night these coaches rolled along at their pace of seven or eight miles an hour. People waited at the cross-roads and citizens hovered about the streets, waiting restlessly to hear the news. The guard, as he handed out his mail bags, told of the decisive victory which had crowned and completed the efforts of the British troops. Then the coachman cracked his whip, the guard's horn gave forth once more its notes of triumph, and the coach rolled away, bearing the thrilling news into other districts. Thus was the intelligence conveyed during the first 30 or 40 years of the century. The telegraph and the express trains have superseded the stage coach and the mail bag, and now the whole world is unrolled before the newspaper reader every day.

---

A GERMAN lately married says: "Id vas yoost so easy as a needle cood valk out mit a camel's eye as to get der behindt vord mit a vomans."

### LIFE INSURANCE WITHIN THE REACH OF ALL.

INSURING one's life, which most people deem a duty, has hitherto been a luxury too costly for people of moderate means. Life insurance companies, in order to accumulate their enormous surplus funds, in many cases amounting to millions of dollars, have fixed their rates of premiums at figures which, we repeat, are prohibitory.

The extravagance of what we may properly call this old system of insurance has driven great numbers of persons to the adoption of a newer and better system of life insurance, within the reach of those to whom it is of the greatest use, to wit, persons of moderate means. This system is strictly mutual, and therefore the safest of any ; since, after providing for the ordinary expenses of economical management, members are only taxed the small sums required from time to time to make up the losses by death ; thus members are not taxed three or four times as much as is necessary in order to establish an enormous surplus fund in which they have no interest beyond the amount of their insurance. We believe in the new plan of life insurance, and have shown our faith by taking policies of insurance in two associations. We will forward all necessary printed information to any reader of the JOURNAL who will take the trouble to write to us on the subject.

**KNIGHT BROTHERS,**

SOLICITORS OF

UNITED STATES AND FOREIGN

**PATENTS,**

**Counsellors in Patent Causes**

AND

**MECHANICAL EXPERTS,**

AGENCIES AT PRINCIPAL FOREIGN CAPITALS.

**No. 633 F Street, N. W., Washington, D. C.;**
**75 West Fourth Street, Cincinnati, Ohio;**
**317 Olive Street, St. Louis, Mo.**

**SEND FOR BOOK OF INSTRUCTIONS.**

### DR. HALL'S PORTABLE SPIROMETER.

NO PHYSICIAN questions the value of the spirometer for exercising and expanding the lungs, and for measuring their capacity. The great difficulty has been to find an instrument that can be compressed into a small compass when not in use, so that the physician or patient may conveniently carry it from place to place as circumstances require. Another obstacle to the more general use of this truly valuable instrument has been its price. The unwieldy spirometer of a few years ago cost from $10 to $20. Dr. Hall's Portable Spirometer sells for $7.50, and will be sent free to any address on receipt of the price by the editor of this journal.

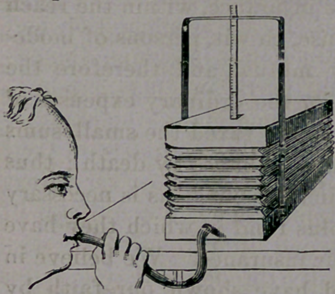

It is 6 by 12 inches, and when closed it stands only 3 inches high, and weighs less than 3 pounds.

This cut represents Dr. Hall's Portable Spirometer, partly inflated, in order to show the manner of using it. When not in use it can be closed together so as to occupy the small space above stated. The scale and guide are hinged, so that they close down on the top of the spirometer, and occupy but little space. The top is of polished black walnut. The brass-work is plated with silver or nickel.

### HOW TO USE THE SPIROMETER.

The instrument should be used only in a perfectly ventilated room, which is entirely free from dust, or else in the open air, away from the dust and smoke and bad odors of the streets of a city. When it is remembered that the capacity of the lungs of a man of medium size is 150 to 250 cubic inches, and that in the act of breathing naturally, or rather as some have acquired the habit of breathing—which is far from natural—the lungs take in only from 20 to 40 cubic inches of air at each inspiration, leaving from 130 to 230 cubic inches of air cells almost always in a dormant condition, the importance of regular and systematic exercise for the lungs will be appreciated. But, like all good things, this sort of lung exercise must be indulged in moderation. Draw a full breath and then blow through the rubber tube into the spirometer; at the same time watch the figures on the scale as it rises. The figures seen on the scale as it passes through the top of the guide indicate the number of cubic inches of air blown into the spirometer.

It is now very generally conceded that mankind start quite evenly on the journey of life, in spite of supposed hereditary tendencies to pulmonary consumption, and that with proper care the average term of life is not shortened by these hereditary tendencies. This being the case, it is far better to enjoy health and long life through means which the naturally robust are not compelled to resort to, than to believe in or lament over the inheritance of ills that do not exist. We believe for all persons who are confined much of the time in doors, particularly in cases where there is a tendency to weak lungs, the regular use of the spirometer in the open air is highly beneficial. Its use just before bedtime is said to induce sleep, by drawing blood from the brain to the lungs.

### CONSTIPATION.

CONSTIPATION becomes alarmingly frequent as people acquire the means of living luxuriously without regular manual labor. It is still more frequent among persons who become so through ignorance of the penalties that follow a want of attention to the regular demands of nature.

When fecal matter is retained in the rectum more than twenty-four hours, it commences its destructive work, notably in two ways. *First*, It is absorbed into the system, and acts as a blood poison. One of the worst results from blood poisoning from constipation is to cause disease of the mind, varying from habitual moroseness to actual insanity. *Second*, By its accumulation and impacting in the lower portion of the rectum it interrupts the circulation of the blood in the adjoining parts, and may produce, from this cause alone, piles, fistula, inflammation of the bladder, disease of the prostate gland, or cancer of the rectum. In fact, most of these diseases are developed in this way. To a very great extent we hold our health and happiness, even our lives, in our own hands. If we sacrifice health and life through our ignorance, or indifference, we pay the penalty through suffering, more severe because more prolonged than instant destruction. From whatever cause, there is, unfortunately, great ignorance in regard to the laws of health among a class of people who are otherwise intelligent. How many parents are there who look carefully to the habits of their children, upon which all that will make life desirable for them so much depends? Cathartics and clysters should not be used in constipation. They increase the trouble.

That which has, perhaps, met with most general favor relates to the diet. Certain foods have been regarded as specifics for this complaint, and particularly "Graham bread." This often induces activity of the bowels, but it ruins digestion like other cathartics, in a manner which we have heretofore fully explained in the pages of this journal. Kneading the bowels has sometimes afforded relief, but it affords no permanent benefit, as a rule. We have, however, found a remedy for constipation and for piles which is always safe, and is attended with no inconvenience or discomfort. We have prescribed it in hundreds of cases. It has usually afforded prompt relief, and many patients who have suffered for years believe that they have been permanently cured of constipation by its use. This remedy is "Nelaton's Suppository." It is prepared and sold by Hall & Ruckel, wholesale druggists, 218 Greenwich street, New York, at 50 cents and $1.00 a box, and is sent free by mail on receipt of price.

